# Using mixed reality to support component restoration of Chinese traditional dwellings: A case study of column bases in Yanling House

**DOI:** 10.1371/journal.pone.0349100

**Published:** 2026-06-12

**Authors:** Binghua Zhang, Yuze Dan

**Affiliations:** 1 School of Architecture and Urban-rural Planning, Fuzhou University, Fuzhou, Fujian, China; 2 Fuzhou Workstation of Key Research Base of National Cultural Heritage Administration (Tsinghua University), Fuzhou University, Fuzhou, Fujian, China; 3 School of Architecture and Urban Planning, Chongqing University, Chongqing, China; 4 Key Laboratory of New Technology for Construction of Cities in Mountain Area, Ministry of Education, Chongqing University, Chongqing, China; Hunan University, CHINA

## Abstract

Due to limited surveying, mapping, and image data, as well as technological constraints, current efforts to restore missing components in traditional Chinese dwellings often result in mismatches with the original appearance. This study employed a mixed-methods approach to evaluate the effectiveness of using mixed reality (MR) to address these challenges. Using an MR system built with Microsoft HoloLens 2 and Trimble Connect, we conducted an on-site experiment with twelve participants focused on column base restoration in the main hall of Yanling House, a typical traditional Chinese dwelling in Jintong Village, Youxi County, Fujian Province. Quantitative questionnaire ratings were utilized to illustrate the effects of MR in this on-site experiment, while qualitative semi-structured interviews provided further elucidation and validation of these effects. The results demonstrate that MR can help participants restore the components in a way that conforms to their original appearance through immersive perceptions, accurate adjustments, and convenient communications, thus providing effective technical support for preserving the unique historical and cultural value of traditional Chinese dwellings.

## Introduction

As an essential part of Chinese cultural heritage, traditional dwellings not only showcase the unique layout, structure, and form of Chinese architecture but also embody particular regional culture and folk art [1]. Nevertheless, in recent years, rapid Chinese urbanization has harmed traditional dwellings, leading to the loss or deterioration of key components, especially column bases, doors, windows, and carved panels. This compromises the structures’ architectural integrity and diminishes their historical and cultural value [2]. Consequently, restoring the components of traditional Chinese dwellings has become extremely urgent, which also accords with international initiatives for cultural heritage conservation, including the Venice Charter, the Convention concerning the Protection of the World Cultural and Natural Heritage, the NARA Document on Authenticity, and the Xi’an Declaration.

However, restoration of the components of traditional Chinese dwellings is challenging. Typically, these components are unique cultural artifacts whose original appearance often primarily persists in the fragmented memories of some residents owing to the lack of accurate surveying, mapping, and image data. Consequently, current restoration work relies on collaborative efforts between cultural heritage conservation experts and residents who once witnessed the original appearance of missing or damaged components [3]. Limited by technologies and tools, the restoration work mainly depends on language, texts, and drawings, which cannot be immersed in the actual architectural environments to evoke the residents’ memories, let alone further communications and adjustments. Therefore, new technology integration into current restoration work is urgently required to support the residents and experts in developing immersive perceptions, accurate adjustments, and convenient communications, thus ensuring that restoration outcomes remain faithful to the original appearance.

The rapid advancement of computer graphics technology has introduced potential ways of coping with these challenges. The earliest computer graphics technology used in cultural heritage conservation was virtual reality (VR). Combined with three-dimensional (3D) reconstruction technologies such as Terrestrial Laser Scanning and Simultaneous Localization and Mapping (SLAM), VR has been primarily used for digital presentation of the restoration results of the components of the cultural heritage [4–7]. Furthermore, through the head-mounted display device, VR provides an effective way for users to experience and understand the restoration results intuitively [8–10]. Nevertheless, VR presents digital restoration results for the components in a way that is entirely separated from the physical architectural environments, which hardly supports immersive perceptions, let alone effective adjustments and communication.

More advanced augmented reality (AR) has been gradually applied to cultural heritage conservation in response to physical architectural environments. AR can bring 3D digital objects into actual environments through mobile computing devices for interactive visualization [11]. In concrete terms, users can view 3D models of the restoration results of the potential components of cultural heritage on site using mobile computing devices [12,13]. Simultaneously, a mobile computing device can act as a tangible interactive interface (TUI) to support users in adjusting 3D models in real time [14,15]. However, limited by the 2D screen of mobile computing devices, the AR display is merely a visual superimposition of virtuality and reality and cannot present real spatial depth [16]. Simultaneously, restricted by the touching interface of mobile computing devices, AR interaction is relatively rough, barely supporting exact controls [11]. Therefore, despite being on-site, AR hardly enables immersive perception, accurate adjustments, or convenient communication.

The latest mixed reality (MR) technology, which integrates the benefits of VR and AR, can create new visual environments in which virtual and real objects coexist and interact in real-time [17]. Compared to an entirely virtual display detached from the physical world, as in the case of VR, MR could bring virtual objects to the real architectural environment for on-site visualization [18,19]. Furthermore, rather than an AR display, which relies on a 2D screen, MR could render a stereoscopic scale for virtual objects and present the spatial relations between them and the real environment [20,21]. It has the potential to support immersive perception. In addition, MR can facilitate real-time interaction based on gaze and gesture, thereby addressing key limitations inherent in VR and AR’s traditional tangible interaction interfaces (such as handles, panels, and screens) [22,23]. Moreover, rather than the mere visual superimposition of AR, MR could achieve a 3D physical interaction (collision or occlusion) between virtuality and reality [24,25]. It could support accurate control and adjustments of the virtual objects. Additionally, by leveraging rapidly developing hardware, different users can immerse themselves in the same MR interactive visualization environment, facilitating intuitive and convenient communication [26,27]. Therefore, MR has the technical capability to address the perception, interaction, and communication limitations of conventional VR and AR technology, thereby ensuring that the restoration results of traditional Chinese dwellings’ components align with historical authenticity.

Currently, MR technology has already witnessed several applications and explorations in related cultural heritage fields. First, MR has been used in the digital preservation and virtual reconstruction of cultural heritage, enabling detailed documentation without physical intervention. By integrating technologies like laser scanning, photogrammetry, and ground-penetrating radar, MR facilitates the creation of accurate 3D models for sites that are damaged, inaccessible, or at risk of deterioration. For instance, Barrile et al. [28] combined these methods to model the “Madonna dei Poveri” church in Italy, capturing both its visible and subterranean structures. Similarly, De Luca et al. [29] developed a mobile MR application utilizing virtual portals to digitally reconstruct the interior of the Basilica of Sant’Elia, enabling users to explore its previously inaccessible spaces. These digital archives serve as vital conservation, research, and virtual restoration resources. They also enable physical replication through 3D printing, providing digital archives and tangible alternatives for fragile artifacts [30–32]. In terms of museum exhibitions and visitor experiences, MR significantly enhances visitor immersion and narrative engagement. It overlays digital information onto physical artifacts, transforming static displays into dynamic storytelling platforms. Li et al. [33] highlighted how MR, primarily through head-mounted displays, greatly enriches visitor immersion. Applications range from allowing users to interact with the digital reconstructions of historical objects, as demonstrated by Paulauskas et al. [34], to exploring submerged cultural sites like coral reefs through photogrammetry-derived models [35]. This approach enhances the accessibility and memorability of heritage by appealing to diverse audiences, including younger generations and international tourists. This appeal is built upon a seamless blend of the physical and virtual [36,37]. Additionally, MR has profoundly transformed educational and cultural learning by providing interactive, collaborative, and experiential environments. It increases student motivation and academic performance by simulating archaeological sites and historical events, offering hands-on experiences that traditional methods cannot [38–39]. Bekele et al. [40] proposed a cloud-based collaborative MR framework for multi-modal cultural learning, enabling multiple users to engage simultaneously with virtual artifacts, strengthening emotional and contextual connections. MR books, combined with mobile applications, help students understand cultural heritage and enhance their cultural identity [41]. Eye-tracking and hand-tracking technologies [42] deepen cross-cultural comprehension. MR bridges the gap between complex history and modern learners, fostering deeper cultural awareness and knowledge retention [43,44]. Furthermore, MR supports innovative collaboration and participation in cultural heritage restoration activities. It enables remote multi-user exploration of heritage sites, allowing both experts and the public to experience locations that are geographically or physically inaccessible, either synchronously or asynchronously [45–47]. Jouan et al. [48] developed a framework through which stakeholders can collaboratively understand cultural heritage values in a virtual space. Moreover, MR aids conservators in overlaying real-time data and spatial guidance on their restoration projects, thus improving accuracy and efficiency [30,49].

In summary, MR has been successfully applied to digital reconstruction, exhibition, education, and public participation in cultural heritage restoration activities. However, MR is primarily used for presenting and interacting with the results of cultural heritage restoration efforts. It does not address the process by which those results are derived or the question of their historical authenticity [33,48]. Our study focuses on utilizing MR to support the collaborative cultural heritage restoration process, facilitating stakeholders’ perception, communication, and decision-making to ensure the authenticity of restoration outcomes.

This study aimed to apply MR to the restoration of components of traditional Chinese dwellings by integrating 3D models of the potential restorative components into real architectural environments to achieve on-site visualization and real-time interaction. This helps participants to ensure that the component restoration results conform to their original appearance through immersive perceptions, accurate adjustments, and convenient communication. Its effectiveness was examined through an on-site experiment using a representative case study and a mixed-methods approach.

## Methods

In line with the study objectives, we selected Yanling House as the restoration case. We used an MR system built with Microsoft HoloLens 2 and Trimble Connect as the technical platform for an on-site experiment involving twelve participants, focused on column base restoration in the house's main hall. We employed a mixed-methods approach to evaluate the effectiveness of MR.

### Ethics statement

This study was reviewed and approved by the Research Ethics Committee of the School of Architecture and Urban-rural Planning, Fuzhou University. All participants provided written informed consent to participate in the study and have their anonymized data published. The individuals in this manuscript have given written informed consent (as outlined in the PLOS consent form) to publish their case details. All procedures were conducted in accordance with the ethical standards of the Declaration of Helsinki.

### Case selection

**Fig 1:**
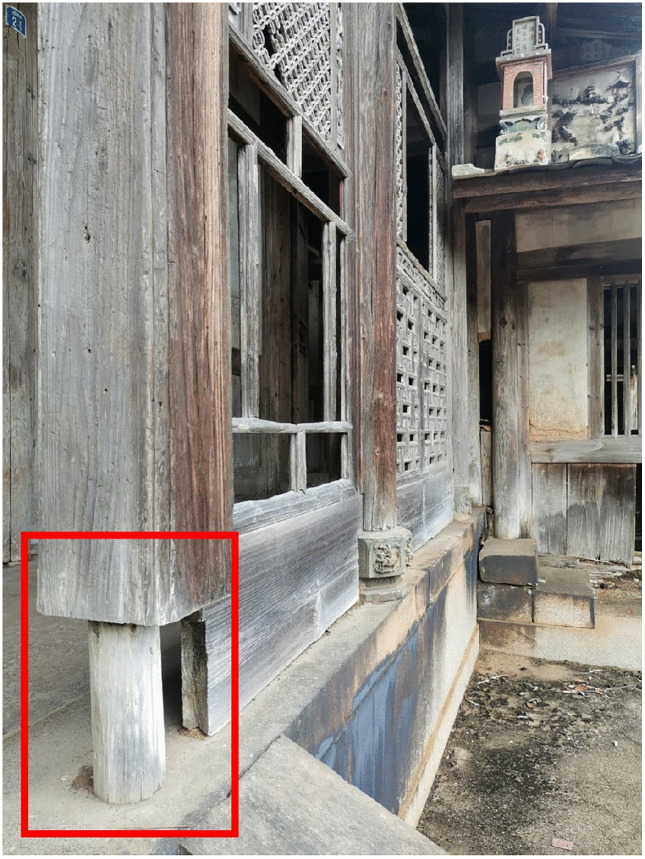
The missing eave column bases of Yanling House’s main hall.

**Fig 2:**
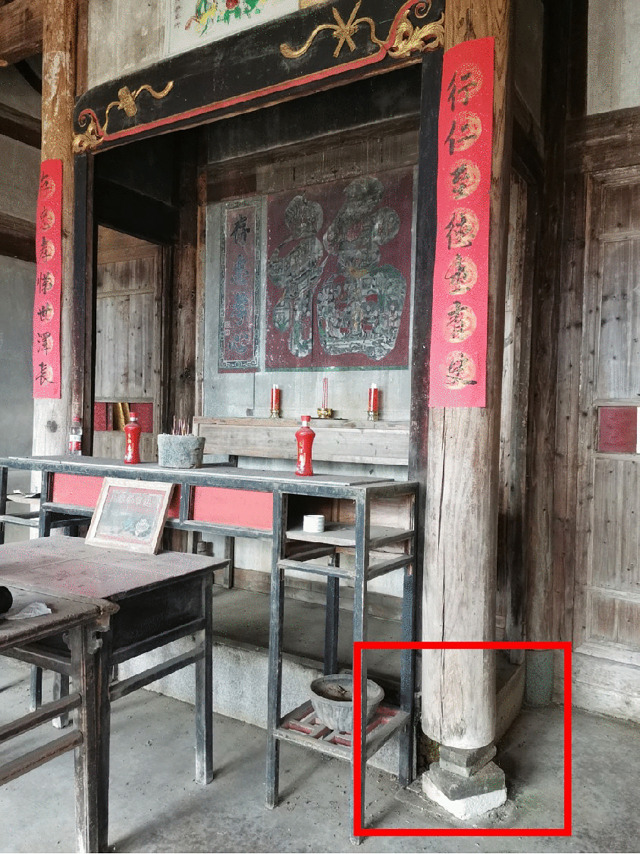
The missing shrine column bases of Yanling House’s main hall.

**Fig 3:**
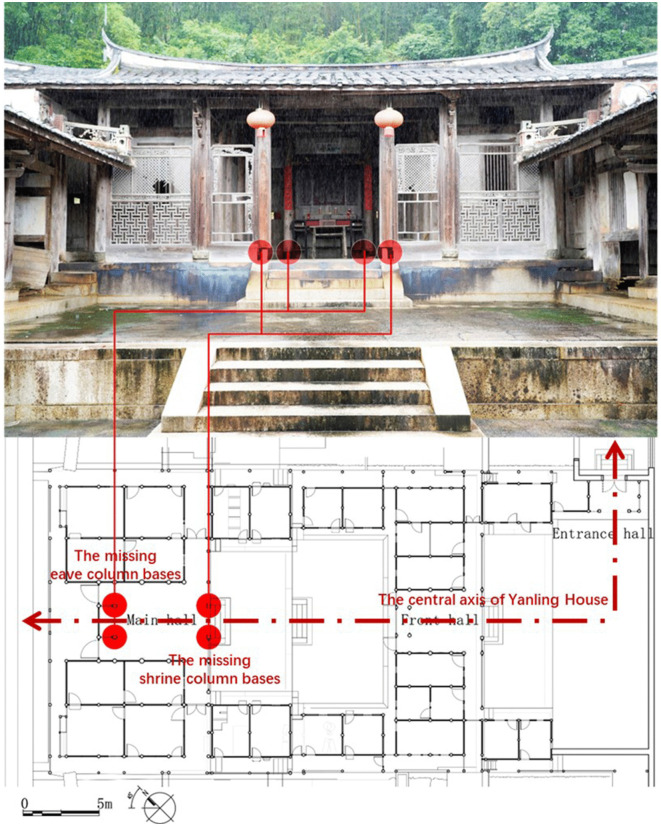
The missing column bases were on the central axis of Yanling House.

We selected Yanling House, a traditional dwelling located in Jingtong Village, Youxi County, Fujian Province, China, as a case study. Yanling House, also known as Shuiweilincuo, was built during the Daoguang period of the Qing Dynasty and is one of the eight most significant traditional dwellings in Youxi County [3]. It was one of the top ten critical discoveries in the third national cultural relics survey conducted by the Chinese State Administration of Cultural Heritage in 2008 [50]. The architectural layout and form of Yanling House are orderly and meaningful. Its components are mainly squares and circles, which adhere to “the heavens being round and the earth being square” concepts in ancient Chinese philosophy. Its decorative patterns are highly regional and predominantly constituted by auspicious symbols (such as propitious animals, plants, and classical stories) from Chinese mythology that signify luck, health, and success. However, at the vital position of Yanling House’s main hall, two pairs of architectural load-bearing components, column bases, were stolen, one after the other, severely affecting its architectural structure and historical and cultural value (Figs 1–3).

**Fig 4:**
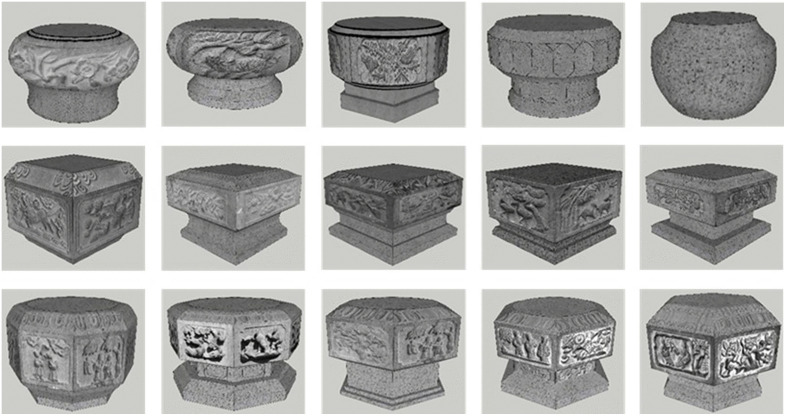
3D models of the potential restorative column bases.

Because the theft occurred a long time ago (more than 20 years) and because of the lack of relevant surveying, mapping, and image data, the original appearance of the column bases of Yanling House’s main hall only existed in the memories of a few residents who had ever seen them. Therefore, cultural heritage conservation experts interviewed these residents in their previous work to acquire fundamental information about the column bases. Following this fundamental information, the experts referenced many documents about the relevant column bases. They performed an analysis and deduction, thus preliminarily restoring a few potential column bases: a circular base, a square base, and eight corrugated bases with different decorative patterns (Fig 4).

This on-site experiment was designed to use MR to place the 3D models of these potential column bases at the damaged positions of Yanling House’s main hall, providing immersive perceptions, evoking the memories of the residents, deepening the analysis and deduction of the experts, and assisting them in making accurate adjustments and convenient on-site communication, thus ensuring that the restoration results of the missing column bases conform to their original appearance.

### The MR system

#### Hardware.

**Fig 5:**
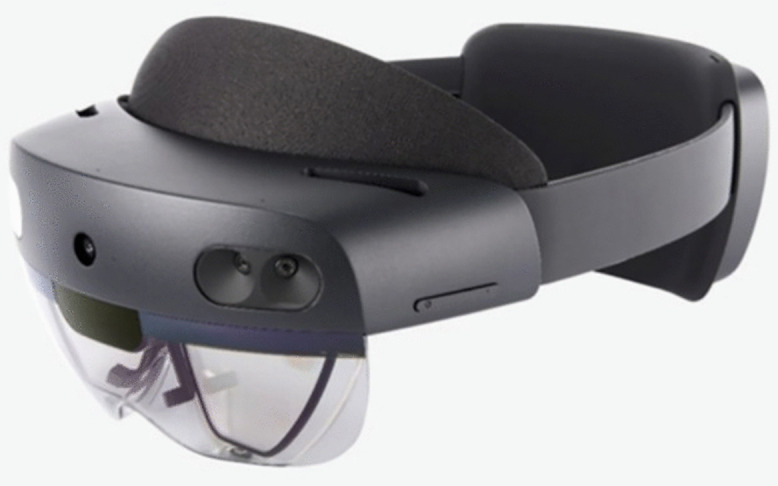
Microsoft HoloLens 2.

In this study, we selected Microsoft HoloLens 2, state-of-the-art MR wearable smart glasses, as the hardware component of the MR system (Fig 5). Microsoft HoloLens 2 contains three computing units: the central processing unit, graphics processing unit, and holographic processing unit, integrated with a variety of sensing elements, such as head tracking, eye tracking, and depth perception sensors, and equipped with display modules that include a light guide prism and miniature projection lens [51]. Therefore, it can obtain environmental information using sensing elements, generate holograms with computing units, and project them into the natural environment using a display module [51].

#### Software.

**Fig 6:**
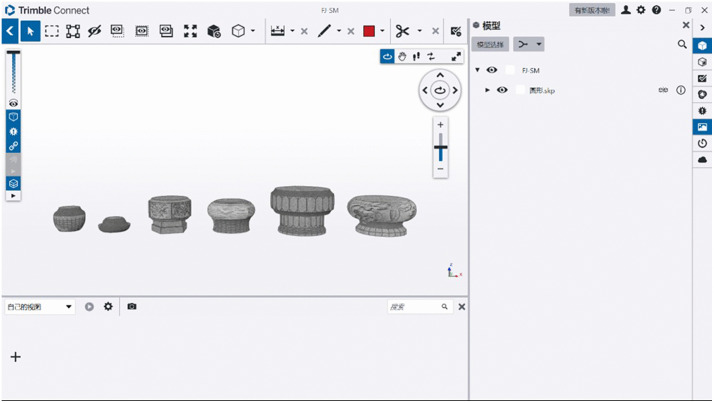
The interface of Trimble Connect.

Trimble Connect software from the official Microsoft HoloLens 2 App Store was used in this study (Fig 6). Trimble Connect is a cloud-based software application that connects different terminals and supports multiformat data storage, viewing, and updating. It can synchronize 3D models from a personal computer (PC) to Microsoft HoloLens 2 for on-site visualization [52]. At the same time, Trimble Connect is equipped with essential restoration and design functions such as moving, rotating, zooming, scaling, and measuring and relies on the sensing elements of Microsoft HoloLens 2 to support real-time interaction for 3D models [52].

**Fig 7:**
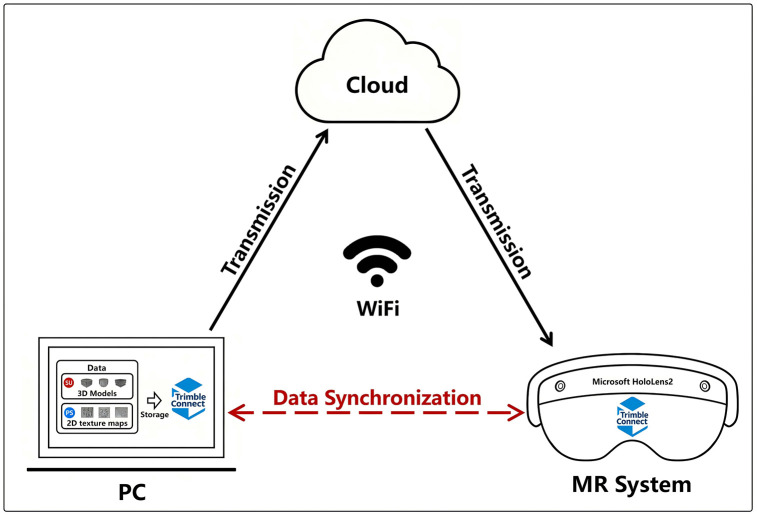
Data synchronization process.

In accordance with the data transmission requirements of the hardware and software, we prepared the corresponding 3D models and 2D texture maps for the potential column bases. Fig 7 shows the 3D models and 2D maps created using Google Sketchup and Adobe Photoshop and stored in Trimble Connect on the PC end. Because Microsoft HoloLens 2 is also equipped with Trimble Connect, it can synchronize data through cloud transmission.

### On-site experiment

On September 24, 2023, between 10:00 AM and 3:00 PM, we conducted an on-site experiment using MR to restore the column bases in Yanling House’s main hall.

#### Experimental participants.

In this experiment, we recruited twelve participants, including six cultural heritage conservation experts and six residents, all of whom participated in previous work on the column base restoration of Yanling House’s main hall. These included six experts from Youxi County Museum, Fuzhou University, and other scientific research institutions, aged 34–63 years old, and six residents aged 40–64 years. In addition, none of the twelve participants had any prior experience with MR usage, ensuring they could neutrally experience the differences in restoration work with and without MR.

Given the practical conditions of the Yanling Hall restoration, we employed targeted sampling to select a small yet highly relevant sample of six residents and six experts to participate in this experiment. These six residents represent the complete population of currently available witnesses who had seen the missing column bases and retained partial memories of their appearance; they are the only individuals still residing in the village with such firsthand experience. The resident group included original homeowners and other village residents. Because the residence was uninhabited and other original villagers who might remember the bases no longer reside in the village and have no means of contact, participants’ perspectives are particularly valuable. The expert group consisted of six professionals who were directly involved in organizing the preliminary restoration work of this traditional dwelling, equipping them with comprehensive knowledge of the local architectural style. Consequently, the qualitative and quantitative feedback provided by both groups held significant value, contributing to a deeper understanding of the restoration process, perceptual experiences, technological advantages, and persistent challenges.

#### Experimental devices.

In addition to the two wearable MR smart glasses, Microsoft HoloLens 2, we used a smartphone (Huawei) and two laptops (Microsoft Surface laptop) in this on-site experiment. Specifically, the smartphone provided a 5G wireless network covering the Yanling House’s main hall through hotspots. In this wireless network, the two Microsoft HoloLens 2 rely on their respective installed software, Trimble Connect, to design the MR system. Microsoft HoloLens 2 was also used as the mobile terminal of the MR system for participants to wear during the experiment. In addition, the two laptops shared the MR scene presented by the two Microsoft HoloLens 2 in real time through the wireless network.

#### Experimental procedures.

**Fig 8:**
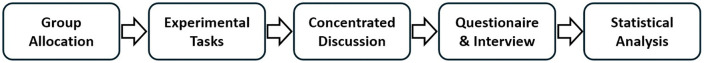
Experimental procedures.

Five experimental procedures were carried out: group allocation, experimental tasks, concentrated discussion, questionnaire and interview, and statistical analysis (Fig 8).

First, we divided the twelve experimental participants into six groups of pairs, each with one resident and one cultural heritage conservation expert.

**Fig 9:**
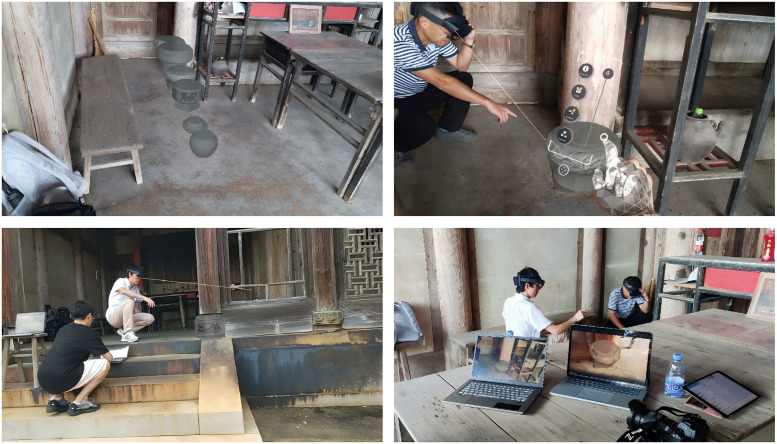
Experimental tasks: on-site restoration work using MR.

According to the grouping order, we gave each group (two participants) 5–10 minutes of usage training to help them learn the basic control skills of the MR system. Each group then used this MR system to conduct 10–15 minutes of on-site restoration work in the actual architectural scene of Yanling House (Fig 9). Specifically, each group’s resident and expert first entered the interactive visualization environment of virtuality and reality coexistence generated by the MR system. Then, through collaboration and communication, they selected the appropriate 3D models of the column bases to be placed at the damaged position of Yanling House’s main hall and made real-time adjustments according to the environmental details.

**Fig 10:**
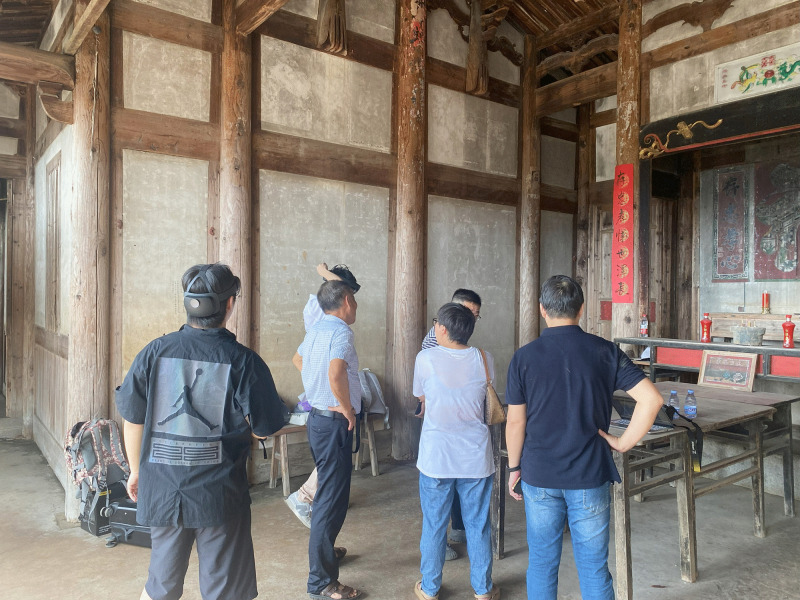
Centralized discussion: Participants discussing the final restoration results.

Subsequently, the twelve participants from the six groups gathered to discuss and determine the final restoration results according to their respective judgments (Fig 10).

**Fig 11:**
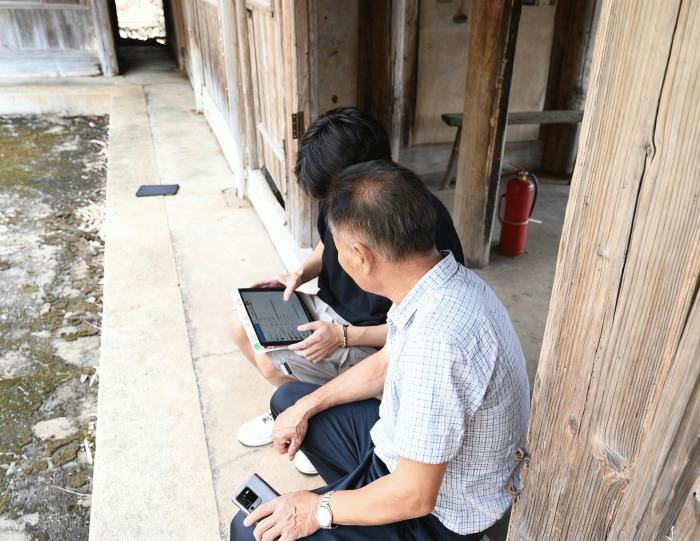
The semi-structured interviews.

**Fig 12:**
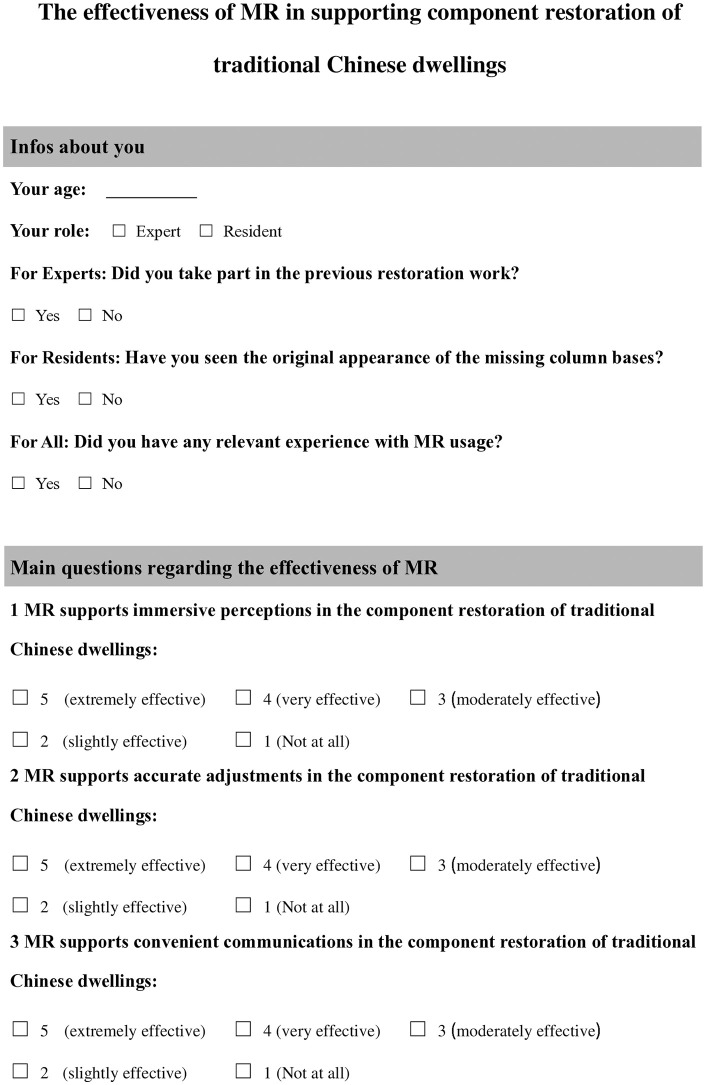
The questionnaire.

Next, we conducted a brief questionnaire followed by a corresponding semi-structured interview with each participant to evaluate the effectiveness of MR in supporting traditional Chinese dwelling component restorations (Fig 11). This questionnaire primarily assessed MR’s effectiveness in facilitating perceptions, adjustment, and communication, with responses rated on a five-point Likert scale (Fig 12).

**Fig 13:**
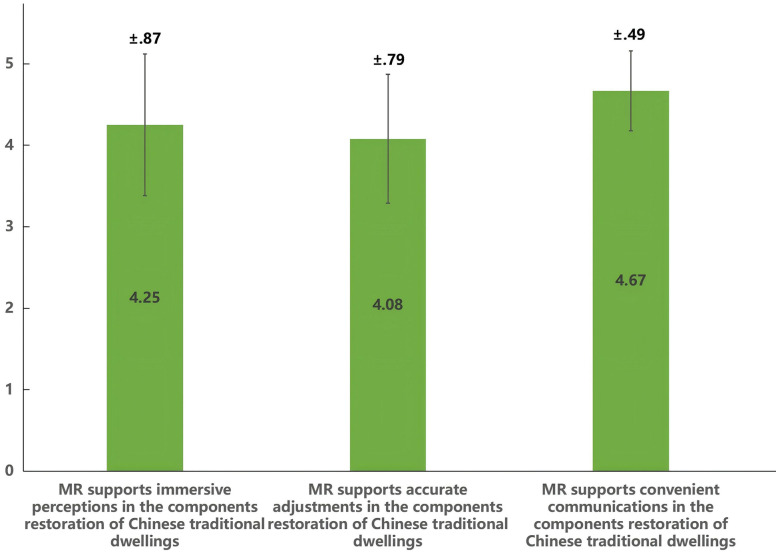
The mean and standard deviation of the ratings for the main contents of the semi-structured interview.

The rating feedback from the questionnaire is presented using descriptive statistics (Fig 13), while the semi-structured interviews provide contextual interpretation and supplementary insights into these ratings. The Kruskal-Wallis test was used to analyze differences in ratings between residents and experts (Table 1), and the Spearman correlation analysis was applied to examine the relations between participants’ age and rating results (Table 2). Owing to the relatively small sample size (12 participants), the results derived from the Kruskal–Wallis test and the Spearman correlation analysis are exploratory in nature. These findings were further elucidated and validated using insights obtained from the semi-structured interviews.

**Table 1:** The Kruskal-Wallis test results.

	Mean ranks of participants’ ratings		Kruskal-Wallis test statistic (*H*)	p-value	Effect Size （*ε²*）
	The residents	The experts			
**MR supports immersive perceptions in the components restoration of Chinese traditional dwellings**	7.50	5.50	1.086	.297	.009
**MR supports accurate adjustments in the components restoration of Chinese traditional dwellings**	6.17	6.83	0.116	.733	−.088
**MR supports convenient communications in the components restoration of Chinese traditional dwellings**	8.00	5.00	1.375	.241	.038

Note. *p* < .05 indicates statistical significance. Effect size *ε²* was calculated as *ε*^*2*^ = (*H* − *k* + 1)/(*n* − *k*), where *H* is the Kruskal-Wallis test statistic, *k* the number of groups, and *n* the total sample size, following Tomczak M and Tomczak E (2014) [53]. Interpretation criteria were: negligible (< .01), small (.01 ≤ *ε²* < .06), medium (.06 ≤ *ε²* < .14), and large (≥ .14), with negative values treated as *ε²* = 0 as mathematical artifacts [53].

**Table 2:** The Spearman correlation analysis results.

		The ratings for “MR supports immersive perceptions in the components restoration of Chinese traditional dwellings”	The ratings for “MR supports accurate adjustments in the components restoration of Chinese traditional dwelling”	The ratings for “MR supports convenient communications in the components restoration of Chinese traditional dwellings”
**Participants’ ages**	**Spearman’s *ρ***	−.171	−0.771	−.256
	***p*-value**	.596	.003	.422
	**95% Confidence Interval (CI)**	[-.659, .452]	[-.954, -.260]	[-.785, .433]

Note. Spearman’s *ρ* with 95% bias-corrected and accelerated (BCa) bootstrapped confidence intervals (CIs) in brackets (5,000 resamples). *p* < .05 is considered statistically significant. Effect size interpretation: |*ρ*| < .10 = negligible, .10–.29 = small, .30–.49 = medium, ≥ .50 = large.

## Results

This section presents the main findings of the on-site experiment. First, we discuss the final restoration results of the missing column bases of the main hall of Yanling House. Subsequently, we describe and analyze the primary results related to perceptions, adjustments, and communication. Specifically, the following three subsections present quantitative questionnaire results to demonstrate MR’s effectiveness in restoring Yanling House’s main hall column base. Next, further qualitative analysis and interpretation based on semi-structured interviews are presented to uncover the causal relations behind the statistical trends.

### Component restoration results

**Fig 14:**
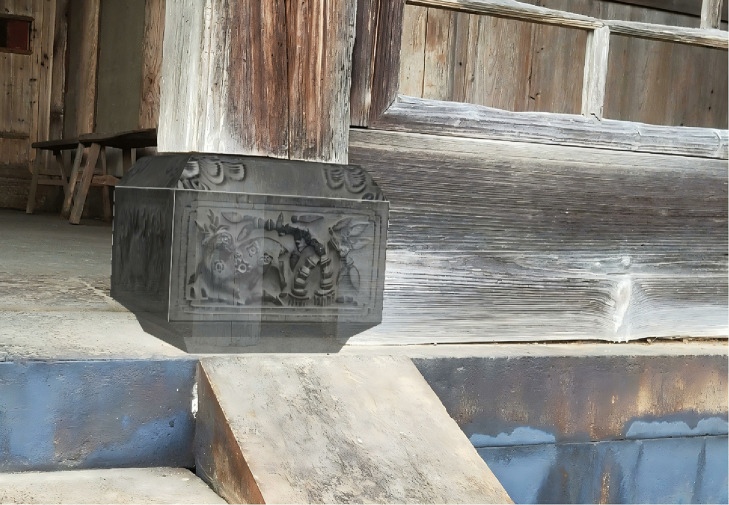
The restoration result of the missing eave column bases of Yanling House’s main hall.

**Fig 15:**
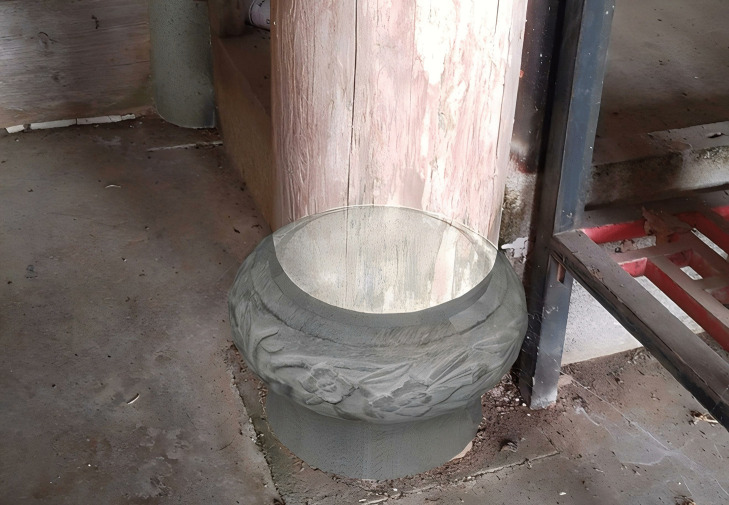
The restoration result of the missing shrine column bases of Yanling House’s main hall.

The on-site experiment showed that by relying on the technical characteristics of MR, 3D models of the potential column bases prepared in advance could be seamlessly integrated into the main hall of Yanling House for on-site 3D visualization and achieve real-time interaction with physical environments and participants. Based on this, participants could have immersive perceptions, make accurate adjustments, experience convenient communication, and accomplish the final restoration results: the missing eave column bases were the square base with a “bamboo deer” pattern (Fig 14); the missing shrine column bases were the round base with a “branched lotus” pattern (Fig 15).

### Immersive perceptions in component restoration using MR

**Fig 16:**
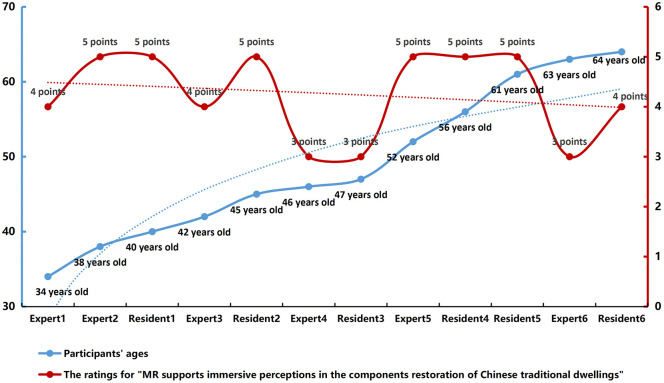
Participants’ ratings for “MR supports immersive perceptions in the component restoration of traditional Chinese dwellings”.

According to the questionnaire survey, the mean and standard deviation of the scores for “MR supports immersive perceptions in the component restoration of traditional Chinese dwellings” were 4.25 and .87, respectively (Fig 13). As shown in Fig 16, most participants gave good feedback: six scored five points, three rated four points, and the others gave relatively neutral scores (three points). Specifically, in the semi-structured interview, almost all participants expressed that MR could not change any details of the actual architectural environments of Yanling House; MR could present the three-dimensional shape, precise scale, and vivid texture of 3D models of potential restorative column bases; and MR could reflect the exact spatial relations between 3D models and architectural environments. Thus, immersive perceptions can be created using MR in the restoration work of the on-site experiment. A few participants pointed out that the current MR system could have been more user-friendly because of its relatively overloaded weight and narrow field of visualization, which somewhat affected their perception and understanding. For instance, a resident shared his experience by saying, “Wearing this device, I can see the virtual column base models placed in the actual environment as if they were real, without having to dismantle or move any structures. However, the device is somewhat heavy, and wearing it for extended periods can be uncomfortable. Also, its field of view is narrow, similar to the framing of a camera.”

In addition, as part of our exploratory quantitative assessment, the Spearman correlation analysis (*p* = .596 > .05, *ρ* = −.171, 95% CI [−.659, .452]) suggested no statistically significant correlation between participants’ ages and their ratings of “MR supports immersive perceptions in the component restoration of traditional Chinese dwellings” in this small sample (Table 2). In the semi-structured interviews, almost all participants responded that MR could create immersive perceptions for them, which strongly supports the view that the improvements in perceptions originated from the MR technology itself and were not associated with the users’ ages.

Moreover, consistent with the exploratory aim of the quantitative analysis, the Kruskal-Wallis test (*H* = 1.086, *p* = .297 > .05, *ε²* = .009 < .01) indicated no statistically significant difference between the residents and cultural heritage conservation experts in the ratings of “MR supports immersive perceptions in the component restoration of traditional Chinese dwellings” in this preliminary investigation (Table 1). In the semi-structured interviews, the residents generally stated that the immersive experiences provided by MR helped them view, recognize, and understand the restoration scene, thus evoking their memories of the missing column bases. For example, a resident recalled, “We have not lived here for ages. I can barely remember what the column bases that got lost twenty or thirty years ago looked like. However, today, with this device on, when we put each virtual restoration plan back into this building, it looks so real. It has helped me slowly remember the shape and size of the original column bases, and even the patterns on them.” At the same time, the experts noted that the immersive MR perceptions facilitated in-depth analysis and deduction regarding the original appearance of column bases under actual environmental conditions. For example, a cultural heritage conservation expert, after personally experiencing it, said, “Previously, when we were doing restorations, we generally relied on experience and compared similar column bases to roughly place one back. However, today, by wearing this device, we can see a 3D, detailed model. We can compare various restoration plans and discuss details on the spot at the construction site. This is a method we have not tried before, which can help us find a restoration plan that is closer to the original appearance and is very helpful for the restoration work.”

## Accurate adjustments in component restoration using MR

**Fig 17:**
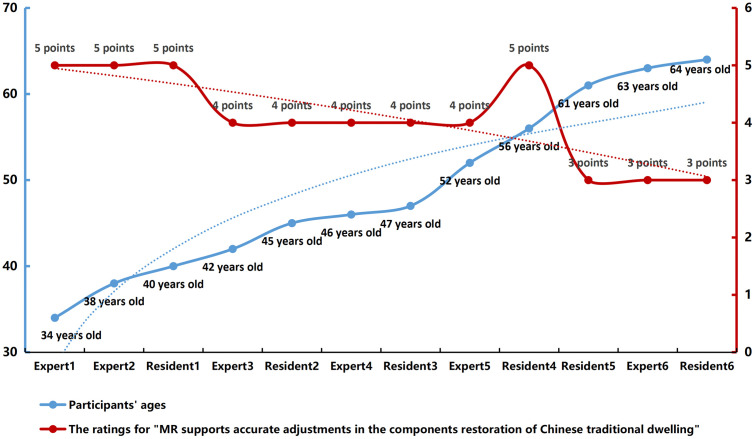
Participants’ ratings for “MR supports accurate adjustments in the component restoration of traditional Chinese dwellings”.

According to the questionnaire survey, the mean and standard deviation for “MR supports accurate adjustments in the component restoration of traditional Chinese dwellings” were 4.08 and .79, respectively (Fig 13). As shown in Fig 17, three participants who were less than 40 years old gave relatively higher ratings (5 points), whereas the feedback of three participants over 60 years old was relatively neutral (3 points). As expected, in this exploratory context, the Spearman correlation analysis (*p* = .003 < .05, *ρ* = −.771, 95% CI [−.954, −.260]) suggested a statistically significant negative association between participants’ ages and their ratings of “MR supports accurate adjustments in the component restoration of traditional Chinese dwellings” in our cohort (Table 2). In the semi-structured interviews, most participants responded that they could use gaze and gestures to accurately adjust the position, size, rotation, and materials of the 3D models of potential restorative column bases based on their real-time judgments. They also stated that these adjustments did not violate the physical laws of the natural world, presenting the physical collision, contact, and occlusion between the digital 3D models and existing architectural environments of Yanling House. Nevertheless, a few older participants indicated that they were not used to this adjustment method depending on gaze and gestures for a short time, even if it could make the restoration more accurate. For example, a cultural heritage conservation expert said, “This technology is quite helpful for the restoration work. However, perhaps because I am getting old and have not been exposed to it before, when adjusting the 3D model on site, my hands and eyes were not yet accustomed to operating this equipment as quickly as young people.”

Furthermore, the exploratory Kruskal-Wallis test (*H* = .116, *p* = .733 > .05) indicated no significant difference between residents and experts in their ratings of “MR supports accurate adjustments in the component restoration of traditional Chinese dwellings” (Table 1). The computed effect size was *ε²* = −.088—a mathematical artifact arising from the formula *ε²* = (*H* – *k* + *1*)/(*n* – *k*) when *H* < *k-1* (*k* = 2 groups, *n* = 12 total), attributable to minimal between-group differences (mean rank: residents = 6.17 vs. experts = 6.83) and small samples (*n* = 6 per group). This negative value has no substantive meaning and was treated as *ε²* = 0—well below the .01 threshold for negligible effects. To address small-sample uncertainty, we computed a 95% CI for *ε²* using non-parametric bootstrap resampling (5000 iterations), yielding [−.10, .45]. This interval includes zero and suggests that even the upper bound of possible effects remains small, confirming that any group differences are statistically and practically negligible.

Semi-structured interviews corroborated these findings: both groups consistently reported mastering the MR system’s basic controls after brief training. Consequently, during on-site experiments, participants from both groups successfully executed accurate restoration adjustments through real-time analysis—consistent with the absence of significant differences in ratings between groups.

### Convenient communication in component restoration using MR

**Fig 18:**
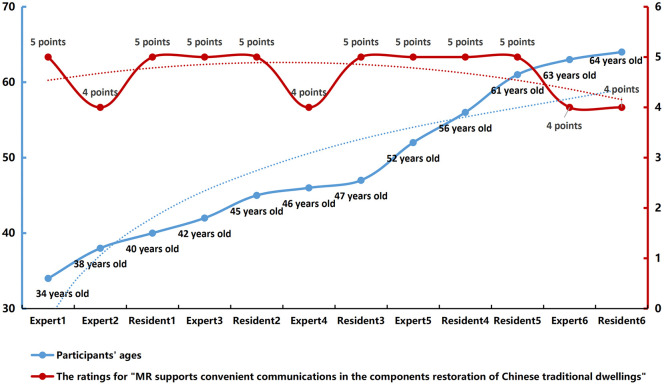
Participants’ ratings for “MR supports participants’ communications in component restoration of traditional Chinese dwellings. ”.

According to the questionnaire survey, the mean and standard deviation of the scores for “MR supports participants’ communications in component restoration of traditional Chinese dwellings” were 4.67 and.49, respectively (Fig 13). As shown in Fig 18, all the experimental participants provided positive feedback. Eight of them thought MR was extremely effective for convenient communication in component restoration, scoring five points, and the other four agreed that MR was very effective, giving four points. Furthermore, in the semi-structured interviews, nearly all participants responded that, in the interactive visualization environments created by MR, where virtual 3D models of potential column bases and the actual Yanling House coexisted, they could express their restoration intentions directly through real-time interaction instead of indirect presentation with language, texts, and drawings. They added that they could acquire others’ restoration suggestions through intuitive 3D visualization, replacing abstract imagination. Therefore, MR facilitated convenient communication among participants in this on-site experiment. For instance, one expert stated, “In previous restoration projects, residents could only provide verbal descriptions without being able to create visual representations. This often made it difficult for others to fully grasp the professional knowledge and experience of experts, leading to significant communication challenges. Now, with this MR system, everyone can participate in the design process on-site, and communication has become considerably more efficient.”

In our exploratory analysis, the Spearman correlation analysis (*p* = .422 > 0.05, *ρ* = −.256, 95% CI [−.758, .433]) suggested no statistically significant correlation between the scores of “MR supports participants’ communication in the component restoration of traditional Chinese dwellings” and participants’ ages within this specific cohort (Table 2). The Kruskal-Wallis test (*H* = 1.375, *p* = .241 > 0.05, *ε*^*2*^ = .038 < .06) suggested no significant difference in ratings between residents and cultural heritage conservation experts in this preliminary assessment (Table 1). Furthermore, the semi-structured interview provided stronger evidence, demonstrating that the facilitated communication among participants stemmed from two technical advantages of MR (3D visualization and real-time interaction), which were independent of the users’ age or other characteristics.

## Discussion

### Benefits

The on-site experiment suggests MR can help participants make immersive perceptions, accurate adjustments, and convenient communication in the current column-based restoration works of the main hall of Yanling House.

Specifically, MR can create 3D interactive visualization environments. In this MR environment, virtual 3D models of the potential restorative components can be located at the actual damaged positions of traditional Chinese dwellings, presenting the correct spatial depth that contributes to related restoration participants making immersive restoration perceptions on site. This not only aids the residents in recalling the original appearance of the missing components but also helps cultural heritage conservation experts analyze and deduce the preliminary restoration proposal under the specific restoration scenario. In addition, in MR environments, the interaction between virtual objects and the real world is not just visual overlaying (like previous TUI); it obeys physical laws and presents the real spatial relations in real time. Through gaze positioning and gesture control in line with human habits, residents can precisely manipulate the 3D models of potential restorative components based on their awakened memories, and experts can make accurate adjustments for the preliminary restoration proposal according to the physical architectural details and real-time judgments. Furthermore, in MR environments, real-time restoration adjustments made by residents or experts can be accurately and instantly conveyed to others through visual information. Thus, convenient and efficient restoration communication can be achieved, which narrows information transmission deviations produced by conventional communication media (such as language, texts, and drawings) and avoids deviation of the restoration results from their original appearance.

Both residents and experts acknowledged in on-site experiments that MR could effectively support the component restoration of traditional Chinese dwellings. On the one hand, MR can aid older residents in retrieving long-term, vague memories. On the other hand, MR enriches the experienced experts’ components restoration methodologies. The 3D interactive visualization environment created by MR can narrow the semantic differences among different knowledge backgrounds. In MR environments, residents can accurately understand professional restoration proposals and express their intentions clearly, allowing them to be totally engaged in the restoration work. To experts, MR can be an effective technical supplementary means for restoring the components of traditional Chinese dwellings when needed.

Compared to existing MR-assisted cultural heritage restoration work focusing on relic reconstruction and museum exhibition [18,20,27,32], this study concentrates on the subtle yet crucial components of cultural heritage—elements often overlooked in existing research but possessing distinct practical and artistic value. Furthermore, unlike current works in which MR is merely utilized for presenting restoration outcomes [19,31,33,42], this study employs MR to assist in the deduction, discussion, and design processes of restoring cultural heritage components, thereby ensuring the authenticity of the restoration results. Moreover, the participatory restoration design advocated in this research leverages MR’s technical capabilities of in-situ visualization and real-time interaction to proactively engage stakeholders (e.g., residents familiar with the house’s original appearance) in the restoration process. This approach enables them to articulate their perspectives and collaboratively participate in decision-making, rather than merely using MR to lower the barriers to comprehension and passively accept restoration outcomes as seen in current practices [40,42,43,48,49]. Finally, rather than a conventional case study of MR application in cultural heritage, this study represents an exploration of novel pathways for cultural heritage restoration. The collaborative restoration methodology facilitated by MR holds significant methodological implications for the preservation and transmission of cultural history in China’s cultural heritage discourse.

### Limitations

Nevertheless, this study had some limitations.

The first limitation concerns the hardware. In this study, we used Microsoft HoloLens 2, a pair of wearable smart glasses that would not have been user-friendly to participants who already wear glasses. Its excessive weight (556 g) and short battery life (2–3 hours) render it unsuitable for prolonged outdoor restoration. In addition, its field of visualization is relatively narrow (43^o^ × 29^o^), which may affect users’ on-site judgments. In addition, unclear imaging under intensive light environments and unstable SLAM under low-light conditions also influence user perceptions, adjustments, and communications to some extent.

The second limitation relates to the network environment. In this study, the MR system used the cloud services and was thus largely influenced by network quality. Unfortunately, traditional Chinese dwellings are usually located far from cities and lack sufficient network base stations. This results in unstable data transmission for component restoration.

The third limitation is the brief duration of the on-site experiment. Due to the relatively simple experimental content, each participant had only 10–15 minutes to experience the MR technology, presenting three key constraints: First, participants may not have been able to fully explore the complete functionality of the MR system, potentially limiting a comprehensive assessment of its perceived support for adjustments and communication during component restoration work. Second, the brief initial experience may have introduced a “novelty effect” bias, where participants’ initial excitement from encountering the new technology could have temporarily inflated their efficacy ratings. Third, the limited exposure time may create ambiguity in interpreting certain results. For example, the observed negative correlation between age and “accurate adjustments” ratings (*p* = .003, *ρ* = −.771) could indicate genuine age-related differences (e.g., technology familiarity, motor dexterity), or result from insufficient training time for older users to achieve proficiency. These ratings may not accurately reflect the technology’s effectiveness under prolonged or repeated use.

The fourth limitation concerns the relatively small sample size. Although the number of participants (six residents and six experts) may limit the generalizability of the findings, it is well aligned with the practical context of component restoration works in traditional Chinese dwellings. The column bases’ unique historical context (lost for decades) resulted in an extremely limited pool of residents with firsthand memory of their original appearance. The six residents represent the complete population of currently available witnesses who had previously seen the missing column bases and retained partial memories of them; other original villagers who might potentially remember them are no longer residing in the village and lack contact information. Therefore, their perspectives are particularly valuable for the restoration of column bases. The six experts were core professionals directly involved in the Yanling Hall restoration project, possessing the requisite expertise to discern the impacts of MR technology. Consequently, this targeted sampling strategy was both contextually appropriate and empirically defensible.

Additionally, a fifth limitation pertains to the evaluation methodology. This study primarily relied on participants’ subjective ratings and qualitative feedback to assess MR’s effectiveness, lacking objective, quantifiable behavioral or performance metrics. Specifically, potential advantages of MR-supported collaboration—such as improvements in communication efficiency (e.g., reduced decision-making time), collaboration quality (e.g., fewer instances of misunderstanding, reduced iterations needed to reach consensus), or task performance—were not precisely measured or recorded. While the qualitative insights provide valuable evidence supporting MR’s role in enhancing understanding and collaboration, the absence of these quantitative data limits our ability to conduct more precise and comparable analyses of MR’s concrete benefits.

Furthermore, this study was not designed to include a controlled experiment. It primarily aimed to employ MR technology to address practical challenges related to discrepancies between restoration outcomes and historical authenticity in the restoration of traditional Chinese dwelling components. The on-site experiment using MR is inherently comparable to current restoration practices conducted without it, thereby serving as an effective control condition. Although a separate control group was not explicitly incorporated, the on-site experiment, combined with a mixed-methods approach, sufficiently demonstrates that MR can be used to effectively address practical challenges and achieve the research objectives. Nonetheless, a controlled experiment would have provided a more intuitive and robust demonstration of the results.

## Conclusion

This study employed MR to support the restoration of traditional Chinese dwelling components, specifically the column bases in the main hall of Yanling House. Six residents and six cultural heritage conservation experts participated in an on-site experiment, guided by a mixed-methods approach to assess MR’s effectiveness. The results show that MR enables on-site visualization and real-time interaction with digital repair models, fostering immersive perception, precise adjustment, and efficient communication among participants. In addressing the absence of unique components with particular historical significance, the full involvement of experts and residents throughout the restoration process, combined with feedback from questionnaires and semi-structured interviews, confirmed that MR offers adequate technical support in restoring the authenticity and integrity of culturally unique architectural elements.

Building upon these promising findings, future research will address the limitations of this study and deepen our understanding of MR’s role in cultural heritage restoration. Specifically, we plan to (1) conduct controlled comparative experiments with larger, more diverse samples across multiple cultural heritage sites to enhance generalizability; (2) undertake longitudinal studies examining sustained MR usage, learning curves, and effectiveness over extended periods and multiple sessions to mitigate potential novelty effects observed in brief exposures; (3) perform rigorous cost-benefit analyses to assess the scalability and practical viability of MR integration into restoration workflows; and (4) investigate optimal MR hardware and software configurations tailored to different restoration scenarios and complexities. Crucially, this future work will prioritize the collection of robust quantitative metrics—such as communication efficiency, iteration cycles to consensus, and error reduction rates—to objectively validate MR’s advantages. Concurrently, the development of more advanced MR tools featuring smoother interaction, flexible storage, and enhanced functionality will continue to support the sustainable preservation and transmission of cultural heritage values through broader application in complex restoration contexts.

## Supporting information

S1 Table Relevant data underlying the findings described in manuscript. (XLSX)
